# But-2-enal 2,4-dinitro­phenyl­hydrazone

**DOI:** 10.1107/S1600536808033254

**Published:** 2008-10-22

**Authors:** Zhi-Gang Yin, Heng-Yu Qian, Yu-Zhen Chen, Jie Hu

**Affiliations:** a Key Laboratory of Surface and Interface Science of Henan, School of Materials and Chemical Engineering, Zhengzhou University of Light Industry, Zhengzhou 450002, People’s Republic of China

## Abstract

In the title compound, C_10_H_10_N_4_O_4_, the but-2-enal chain is almost planar, the largest deviation from the mean plane being 0.013 (1) Å, and this plane makes a dihedral angle of 9.95 (24)° with the benzene ring,. Of the two nitro groups, one is twisted with respect to the benzene ring, making a dihedral angle of 5.7 (1)°, whereas the other is nearly in the plane of the benzene ring, with a twist angle of only 0.7 (1)°. This difference is related to the occurence of an intra­molecular N—H⋯O hydrogen bond with the O atom of the less twisted nitro group. The NH group is also involved in a weak inter­action with the same O atom of a symmetry-related mol­ecule, thus forming a pseudo inversion dimer.

## Related literature

For general background, see: Okabe *et al.* (1993 ▶). For related structures, see: Bolte & Dill (1998 ▶); Ohba (1996 ▶). 
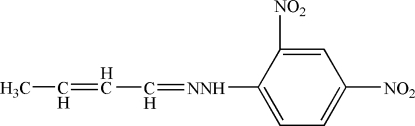


## Experimental

### 

#### Crystal data

C_10_H_10_N_4_O_4_
*M* *_r_* = 250.22
Monoclinic, 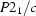

*a* = 4.6699 (8) Å
*b* = 13.188 (2) Å
*c* = 18.880 (3) Å
β = 92.565 (3)°
*V* = 1161.6 (3) Å^3^
*Z* = 4
Mo *K*α radiation
μ = 0.11 mm^−1^
*T* = 293 (2) K
0.27 × 0.23 × 0.23 mm

#### Data collection

Bruker SMART APEX CCD area-detector diffractometer
Absorption correction: multi-scan (*SADABS*; Bruker, 1998 ▶) *T*_min_ = 0.972, *T*_max_ = 0.976
9152 measured reflections
2458 independent reflections
1326 reflections with *I* > 2σ(*I*)
*R*_int_ = 0.039

#### Refinement

*R*[*F* ^2^ > 2σ(*F* ^2^)] = 0.042
*wR*(*F* ^2^) = 0.111
*S* = 0.95
2458 reflections
164 parameters
H-atom parameters constrained
Δρ_max_ = 0.16 e Å^−3^
Δρ_min_ = −0.11 e Å^−3^

### 

Data collection: *SMART* (Bruker, 1998 ▶); cell refinement: *SAINT* (Bruker, 1998 ▶); data reduction: *SAINT*; program(s) used to solve structure: *SHELXS97* (Sheldrick, 2008 ▶); program(s) used to refine structure: *SHELXL97* (Sheldrick, 2008 ▶); molecular graphics: *ORTEPIII* (Burnett & Johnson, 1996 ▶), *ORTEP-3 for Windows* (Farrugia, 1997 ▶) and *PLATON* (Spek, 2003 ▶); software used to prepare material for publication: *SHELXL97*.

## Supplementary Material

Crystal structure: contains datablocks global, I. DOI: 10.1107/S1600536808033254/dn2390sup1.cif

Structure factors: contains datablocks I. DOI: 10.1107/S1600536808033254/dn2390Isup2.hkl

Additional supplementary materials:  crystallographic information; 3D view; checkCIF report

## Figures and Tables

**Table 1:** Hydrogen-bond geometry (Å, °)

*D*—H⋯*A*	*D*—H	H⋯*A*	*D*⋯*A*	*D*—H⋯*A*
N2—H2⋯O1	0.86	2.02	2.6314 (18)	127
N2—H2⋯O1^i^	0.86	2.52	3.331 (2)	159

Symmetry code: (i) 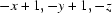.

**Fig. 1.**
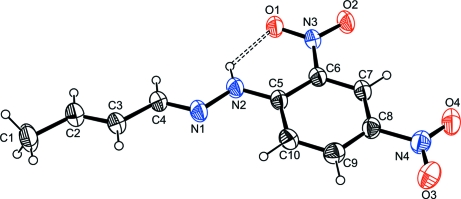
Molecular view of (I) with the atom-labeling scheme. Displacement ellipsoids are drawn at the 30% probability level. H atoms are presented as small spheres of arbitrary radii. Intramolecular H bond is shown as dashed line.

**Fig. 2.**
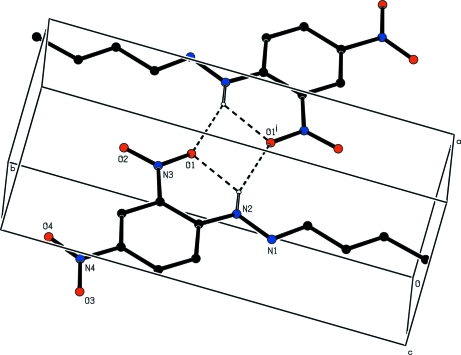
Partial packing view showing the formation of pseudo dimer through weak intermolecular N-H···O hydrogen bonds which are shown as dashed lines. H atoms not involved in hydrogen bondings have been omitted for clarity.[Symmetry code: (i) -x+1, -y+1, -z]

## supplementary crystallographic information

### Comment

2,4-Dinitrophenylhydrazine has applications in organic synthesis and some of its derivatives have been shown to be potentially DNA-damaging and mutagenic agents (Okabe *et al.*, 1993). Some phenylhydrazone derivatives have been synthesized in our laboratory. As part of our work, we report the synthesis and crystal structure of the title compound(I).

In the title compound, the but-2-enal chain is planar with the largest deviation from the mean plane being 0.013 (1)Å at C1. This plane makes a dihedral angle of 9.95 (24)° with the benzene ring, so the whole molecule is roughly planar (Fig. 1). Of the the two nitro groups, one is twisted with respect to the benzene ring making a dihedral angle of 5.7 (1)° whereas the other is nearly in the plane of the benzene ring with a twist angle of only 0.7 (1)°. This difference is related to the occurence of an intramolecular hydrogen N-H···bond with the O atom of the less twisted nitro group (Table 1). The NH is also in weak intermolecular interaction with the same O atom of a symmetry related molecule building a pseudo dimer (Table 1, Fig. 2).

Bond lengths and bond angles are consistent with those of other dinitrophenylhydrazone derivatives(Ohba,1996; Bolte & Dill, 1998)

### Experimental

2,4-Dinitrophenylhydrazine (1 mmol, 0.198 g) was dissolved in anhydrous methanol, H_2_SO_4_ (98% 0.5 ml) was added to this, the mixture was stirred for several minitutes at 351 K, but-2-enal (1 mmol 0.070 g) in methanol (8 ml) was added dropwise and the mixture was stirred at refluxing temperature for 2 h. The product was isolated and recrystallized in methanol, red single crystals of (I) was obtained after two weeks.

### Refinement

H atoms were placed in calculated position and treated as riding with C—H= 0.93Å (aromatic), 0.96Å(methyl) and N-H= 0.86\%A with *U*_iso_(H)=1.2*U*_eq_(C,N) or *U*_iso_(H)=1.5*U*_eq_(methyl).

### Crystal data

**Table:**

C_10_H_10_N_4_O_4_	*F*(000) = 520
*M**_r_* = 250.22	*D*_x_ = 1.432 Mg m^−^^3^
Monoclinic, *P*2_1_/*c*	Mo *K*α radiation, λ = 0.71073 Å
Hall symbol: -P 2ybc	Cell parameters from 1673 reflections
*a* = 4.6699 (8) Å	θ = 2.1–25.5°
*b* = 13.188 (2) Å	µ = 0.11 mm^−^^1^
*c* = 18.880 (3) Å	*T* = 293 K
β = 92.565 (3)°	Block, red
*V* = 1161.6 (3) Å^3^	0.27 × 0.23 × 0.23 mm
*Z* = 4	

### Data collection

**Table:**

Bruker SMART APEX CCD area-detector diffractometer	2458 independent reflections
Radiation source: sealed tube	1326 reflections with *I* > 2σ(*I*)
graphite	*R*_int_ = 0.039
φ and ω scans	θ_max_ = 27.0°, θ_min_ = 2.7°
Absorption correction: multi-scan (SADABS; Bruker, 1998)	*h* = −5→5
*T*_min_ = 0.972, *T*_max_ = 0.976	*k* = −16→16
9152 measured reflections	*l* = −24→24

### Refinement

**Table:**

Refinement on *F*^2^	Primary atom site location: structure-invariant direct methods
Least-squares matrix: full	Secondary atom site location: difference Fourier map
*R*[*F*^2^ > 2σ(*F*^2^)] = 0.042	Hydrogen site location: inferred from neighbouring sites
*wR*(*F*^2^) = 0.111	H-atom parameters constrained
*S* = 0.95	*w* = 1/[σ^2^(*F*_o_^2^) + (0.0552*P*)^2^] where *P* = (*F*_o_^2^ + 2*F*_c_^2^)/3
2458 reflections	(Δ/σ)_max_ < 0.001
164 parameters	Δρ_max_ = 0.16 e Å^−^^3^
0 restraints	Δρ_min_ = −0.11 e Å^−^^3^

### Special details

**Table:**

Geometry. All esds (except the esd in the dihedral angle between two l.s. planes) are estimated using the full covariance matrix. The cell esds are taken into account individually in the estimation of esds in distances, angles and torsion angles; correlations between esds in cell parameters are only used when they are defined by crystal symmetry. An approximate (isotropic) treatment of cell esds is used for estimating esds involving l.s. planes.
Refinement. Refinement of *F*^2^ against ALL reflections. The weighted *R*-factor *wR* and goodness of fit *S* are based on *F*^2^, conventional *R*-factors *R* are based on *F*, with *F* set to zero for negative *F*^2^. The threshold expression of *F*^2^ > σ(*F*^2^) is used only for calculating *R*-factors(gt) *etc*. and is not relevant to the choice of reflections for refinement. *R*-factors based on *F*^2^ are statistically about twice as large as those based on *F*, and *R*- factors based on ALL data will be even larger.

### Fractional atomic coordinates and isotropic or equivalent isotropic displacement parameters (Å^2^)

**Table:**

	*x*	*y*	*z*	*U*_iso_*/*U*_eq_	
N1	0.0380 (3)	0.35052 (10)	0.11439 (8)	0.0607 (4)	
N2	0.1292 (3)	0.44685 (9)	0.09740 (8)	0.0563 (4)	
H2	0.2613	0.4552	0.0676	0.068*	
N3	0.2785 (3)	0.65667 (11)	0.05938 (8)	0.0554 (4)	
N4	−0.3915 (4)	0.77803 (13)	0.22849 (9)	0.0673 (5)	
O1	0.4042 (3)	0.58766 (9)	0.02881 (7)	0.0693 (4)	
O2	0.3266 (3)	0.74610 (9)	0.04720 (8)	0.0761 (4)	
O3	−0.5851 (3)	0.75891 (12)	0.26884 (9)	0.0872 (5)	
O4	−0.3125 (3)	0.86398 (12)	0.21507 (9)	0.0909 (5)	
C1	0.1822 (6)	−0.01443 (14)	0.09371 (14)	0.1054 (9)	
H1A	0.0157	−0.0191	0.1216	0.158*	
H1B	0.3428	−0.0449	0.1191	0.158*	
H1C	0.1469	−0.0492	0.0495	0.158*	
C2	0.2468 (5)	0.09525 (15)	0.07954 (12)	0.0770 (6)	
H22	0.4003	0.1089	0.0513	0.092*	
C3	0.1060 (5)	0.17380 (13)	0.10341 (10)	0.0651 (5)	
H3	−0.0512	0.1615	0.1307	0.078*	
C4	0.1811 (4)	0.27691 (12)	0.08969 (10)	0.0585 (5)	
H4	0.3370	0.2906	0.0623	0.070*	
C5	0.0079 (4)	0.52752 (12)	0.12821 (9)	0.0497 (4)	
C6	0.0733 (4)	0.63020 (12)	0.11150 (9)	0.0506 (4)	
C7	−0.0580 (4)	0.71065 (13)	0.14479 (10)	0.0539 (5)	
H7	−0.0131	0.7771	0.1330	0.065*	
C8	−0.2534 (4)	0.69186 (13)	0.19495 (10)	0.0556 (5)	
C9	−0.3232 (4)	0.59241 (15)	0.21334 (10)	0.0603 (5)	
H9	−0.4562	0.5806	0.2476	0.072*	
C10	−0.1960 (4)	0.51299 (13)	0.18104 (10)	0.0582 (5)	
H10	−0.2439	0.4473	0.1939	0.070*	

### Atomic displacement parameters (Å^2^)

**Table:**

	*U*^11^	*U*^22^	*U*^33^	*U*^12^	*U*^13^	*U*^23^
N1	0.0727 (10)	0.0384 (8)	0.0716 (10)	−0.0054 (7)	0.0084 (8)	0.0014 (7)
N2	0.0651 (9)	0.0405 (8)	0.0639 (9)	−0.0042 (7)	0.0116 (7)	0.0008 (7)
N3	0.0681 (10)	0.0380 (8)	0.0602 (9)	0.0009 (7)	0.0032 (8)	0.0008 (7)
N4	0.0677 (11)	0.0668 (12)	0.0670 (11)	0.0122 (9)	−0.0015 (9)	−0.0095 (9)
O1	0.0902 (10)	0.0448 (7)	0.0747 (9)	0.0027 (7)	0.0259 (7)	−0.0002 (6)
O2	0.1022 (11)	0.0386 (7)	0.0894 (10)	−0.0035 (7)	0.0259 (8)	0.0066 (6)
O3	0.0782 (10)	0.0913 (11)	0.0937 (11)	0.0151 (8)	0.0195 (9)	−0.0164 (8)
O4	0.1150 (13)	0.0545 (9)	0.1047 (12)	0.0163 (9)	0.0220 (10)	−0.0058 (8)
C1	0.162 (2)	0.0418 (11)	0.110 (2)	0.0019 (14)	−0.0186 (17)	0.0004 (12)
C2	0.1028 (17)	0.0469 (11)	0.0809 (14)	0.0004 (11)	0.0013 (12)	−0.0004 (10)
C3	0.0838 (14)	0.0428 (10)	0.0687 (12)	−0.0059 (9)	0.0057 (10)	0.0026 (9)
C4	0.0724 (12)	0.0427 (10)	0.0606 (11)	−0.0040 (9)	0.0060 (9)	0.0011 (8)
C5	0.0527 (10)	0.0394 (9)	0.0564 (11)	−0.0010 (8)	−0.0037 (8)	−0.0010 (7)
C6	0.0528 (10)	0.0445 (9)	0.0543 (10)	−0.0006 (8)	−0.0001 (9)	0.0012 (8)
C7	0.0572 (11)	0.0406 (9)	0.0632 (11)	0.0014 (8)	−0.0062 (9)	−0.0002 (8)
C8	0.0548 (11)	0.0519 (11)	0.0596 (11)	0.0098 (9)	−0.0014 (9)	−0.0042 (9)
C9	0.0545 (11)	0.0661 (12)	0.0605 (12)	0.0025 (9)	0.0051 (9)	−0.0002 (9)
C10	0.0622 (11)	0.0474 (10)	0.0649 (12)	−0.0042 (9)	0.0039 (9)	0.0056 (9)

### Geometric parameters (Å, °)

**Table:**

N1—C4	1.278 (2)	C2—C3	1.317 (3)
N1—N2	1.3821 (17)	C2—H22	0.9300
N2—C5	1.349 (2)	C3—C4	1.431 (2)
N2—H2	0.8600	C3—H3	0.9300
N3—O2	1.2245 (17)	C4—H4	0.9300
N3—O1	1.2396 (17)	C5—C10	1.422 (3)
N3—C6	1.446 (2)	C5—C6	1.427 (2)
N4—O4	1.2221 (19)	C6—C7	1.390 (2)
N4—O3	1.234 (2)	C7—C8	1.367 (3)
N4—C8	1.465 (2)	C7—H7	0.9300
C1—C2	1.504 (3)	C8—C9	1.399 (3)
C1—H1A	0.9600	C9—C10	1.362 (2)
C1—H1B	0.9600	C9—H9	0.9300
C1—H1C	0.9600	C10—H10	0.9300
			
C4—N1—N2	116.23 (15)	N1—C4—C3	121.30 (18)
C5—N2—N1	119.02 (14)	N1—C4—H4	119.4
C5—N2—H2	120.5	C3—C4—H4	119.4
N1—N2—H2	120.5	N2—C5—C10	120.21 (15)
O2—N3—O1	121.65 (15)	N2—C5—C6	123.70 (16)
O2—N3—C6	119.54 (15)	C10—C5—C6	116.08 (16)
O1—N3—C6	118.80 (14)	C7—C6—C5	121.43 (16)
O4—N4—O3	123.62 (17)	C7—C6—N3	116.26 (15)
O4—N4—C8	119.16 (19)	C5—C6—N3	122.30 (15)
O3—N4—C8	117.21 (17)	C8—C7—C6	119.78 (16)
C2—C1—H1A	109.5	C8—C7—H7	120.1
C2—C1—H1B	109.5	C6—C7—H7	120.1
H1A—C1—H1B	109.5	C7—C8—C9	120.80 (17)
C2—C1—H1C	109.5	C7—C8—N4	118.64 (17)
H1A—C1—H1C	109.5	C9—C8—N4	120.55 (18)
H1B—C1—H1C	109.5	C10—C9—C8	119.93 (18)
C3—C2—C1	126.1 (2)	C10—C9—H9	120.0
C3—C2—H22	117.0	C8—C9—H9	120.0
C1—C2—H22	117.0	C9—C10—C5	121.97 (16)
C2—C3—C4	123.7 (2)	C9—C10—H10	119.0
C2—C3—H3	118.1	C5—C10—H10	119.0
C4—C3—H3	118.1		
			
C5—N2—N1—C4	−172.68 (16)	N1—C4—C3—C2	−179.7 (2)
N2—N1—C4—C3	−179.67 (15)	C4—C3—C2—C1	178.41 (19)

### Hydrogen-bond geometry (Å, °)

**Table:**

*D*—H···*A*	*D*—H	H···*A*	*D*···*A*	*D*—H···*A*
N2—H2···O1	0.86	2.02	2.6314 (18)	127
N2—H2···O1^i^	0.86	2.52	3.331 (2)	159

Symmetry codes: (i) −*x*+1, −*y*+1, −*z*.
